# Multicytokine-producing CD4^+^ T cells characterize the livers of patients with NASH

**DOI:** 10.1172/jci.insight.153831

**Published:** 2023-01-10

**Authors:** Anna Woestemeier, Pasquale Scognamiglio, Yu Zhao, Jonas Wagner, Franziska Muscate, Christian Casar, Francesco Siracusa, Filippo Cortesi, Theodora Agalioti, Simone Müller, Adrian Sagebiel, Leonie Konczalla, Ramez Wahib, Karl-Frederick Karstens, Anastasios D. Giannou, Anna Duprée, Stefan Wolter, Milagros N. Wong, Anne K. Mühlig, Agata A. Bielecka, Vikas Bansal, Tianran Zhang, Oliver Mann, Victor G. Puelles, Tobias B. Huber, Ansgar W. Lohse, Jakob R. Izbicki, Noah W. Palm, Stefan Bonn, Samuel Huber, Nicola Gagliani

**Affiliations:** 1 Department for General, Visceral and Thoracic Surgery,; 2 Institute of Medical Systems Biology, Center for Biomedical AI (bAIome), Center for Molecular Neurobiology (ZMNH),; 3 I Department of Medicine,; 4 Bioinformatics Core, and; 5 III. Department of Medicine, University Medical Center Hamburg-Eppendorf, Hamburg, Germany.; 6 Department of Clinical Medicine, Aarhus University, Aarhus, Denmark.; 7 Department of Pathology, Aarhus University Hospital, Aarhus, Denmark.; 8 University’s Children Hospital, UKE Hamburg, Hamburg, Germany.; 9 Department of Immunobiology, School of Medicine, Yale University, New Haven, Connecticut, USA.; 10 Immunology and Allergy Unit, Department of Medicine, Solna, Karolinska Institute and University Hospital, Stockholm, Sweden.

**Keywords:** Hepatology, Immunology, Fibrosis, T cells

## Abstract

A role of CD4^+^ T cells during the progression from nonalcoholic fatty liver disease (NAFLD) to nonalcoholic steatohepatitis (NASH) has been suggested, but which polarization state of these cells characterizes this progression and the development of fibrosis remain unclear. In addition, a gut-liver axis has been suggested to play a role in NASH, but the role of CD4^+^ T cells in this axis has just begun to be investigated. Combining single-cell RNA sequencing and multiple-parameter flow cytometry, we provide the first cell atlas to our knowledge focused on liver-infiltrating CD4^+^ T cells in patients with NAFLD and NASH, showing that NASH is characterized by a population of multicytokine-producing CD4^+^ T cells. Among these cells, only those with a Th17 polarization state were enriched in patients with advanced fibrosis. In parallel, we observed that *Bacteroides* appeared to be enriched in the intestine of NASH patients and to correlate with the frequency of multicytokine-producing CD4^+^ T cells. In short, we deliver a CD4^+^ T cell atlas of NAFLD and NASH, providing the rationale to target CD4^+^ T cells with a Th17 polarization state to block fibrosis development. Finally, our data offer an early indication to test whether multicytokine-producing CD4^+^ T cells are part of the gut-liver axis characterizing NASH.

## Introduction

Nonalcoholic fatty liver disease (NAFLD) is the leading cause of chronic liver disease in the Western world (1, 2). NAFLD can progress to nonalcoholic steatohepatitis (NASH) (3) and approximately 40% of NASH patients inevitably develop severe complications such as fibrosis, cirrhosis, and hepatocellular carcinoma (1, 2). NAFLD and NASH are probably not 2 distinct diseases, but rather a continuum characterized by a progressive inflammatory response. However, the etiology underlying this progression and ultimately the development of fibrosis, a major determinant of mortality (2), remains largely unclear. It is worth noticing that there is not one single drug approved by the Food and Drug Administration (FDA) or European Medicines Agency (EMA) for the treatment of NASH.

Previous mouse studies suggest a role of CD4^+^ T cells in the inflammatory progression from NAFLD to NASH (4, 5). Human studies associated the frequency of different CD4^+^ T cell populations, such as Th1, Th2, Th17, and Foxp3^+^ Treg cells to the immunopathogenesis of NASH (6–8). While these previous studies provided fundamental indications for the potential role of CD4^+^ T cells in NASH, they were based on a supervised analysis that classifies CD4^+^ T cells on the basis of a small number of preselected cytokines. Therefore, these studies did not fully describe the complexity of human T cell biology and their potential implication in NASH.

It is known that CD4^+^ T cell composition and function can be altered by the intestinal microbiota (9). There is increasing evidence not only that the intestinal microbiota might influence intrahepatic T cells, but also that it plays a crucial role in the pathogenesis of liver diseases, including NASH (10–12). By profiling the stool microbiome, it was shown that the abundance of some bacteria, such as *Bacteroides*, was more elevated in NASH patients than in healthy individuals (12, 13). Moreover, bacterial DNA was found in the liver of NAFLD patients (14) and the liver microbial composition differed between morbidly obese and non–morbidly obese NAFLD patients (14).

On the basis of all this, a connection between the intestine and the liver, i.e., the gut-liver axis, has been proposed for the progression from NAFLD to NASH. However, a study analyzing the immune system and the tissue-specific microbiota composition within the same patient cohort remains to be performed. Besides this, previous studies were limited in that they compared either NASH or NAFLD patients with healthy individuals and were thus unable to describe the developmental spectrum covering NAFLD to NASH progression (12–14).

Here, combining single-cell RNA sequencing (scRNA-seq), multiple-parameter flow cytometric analysis, and tissue-derived microbiome sequencing from liver and intestinal samples, our study reveals the complete CD4**^+^** T cell landscape and the microbial composition in liver and intestine of NAFLD and NASH patients.

## Results

### Characterization of 8 distinct subsets of liver-infiltrating CD4^+^ T cells in NAFLD patients.

**Figure 1:**
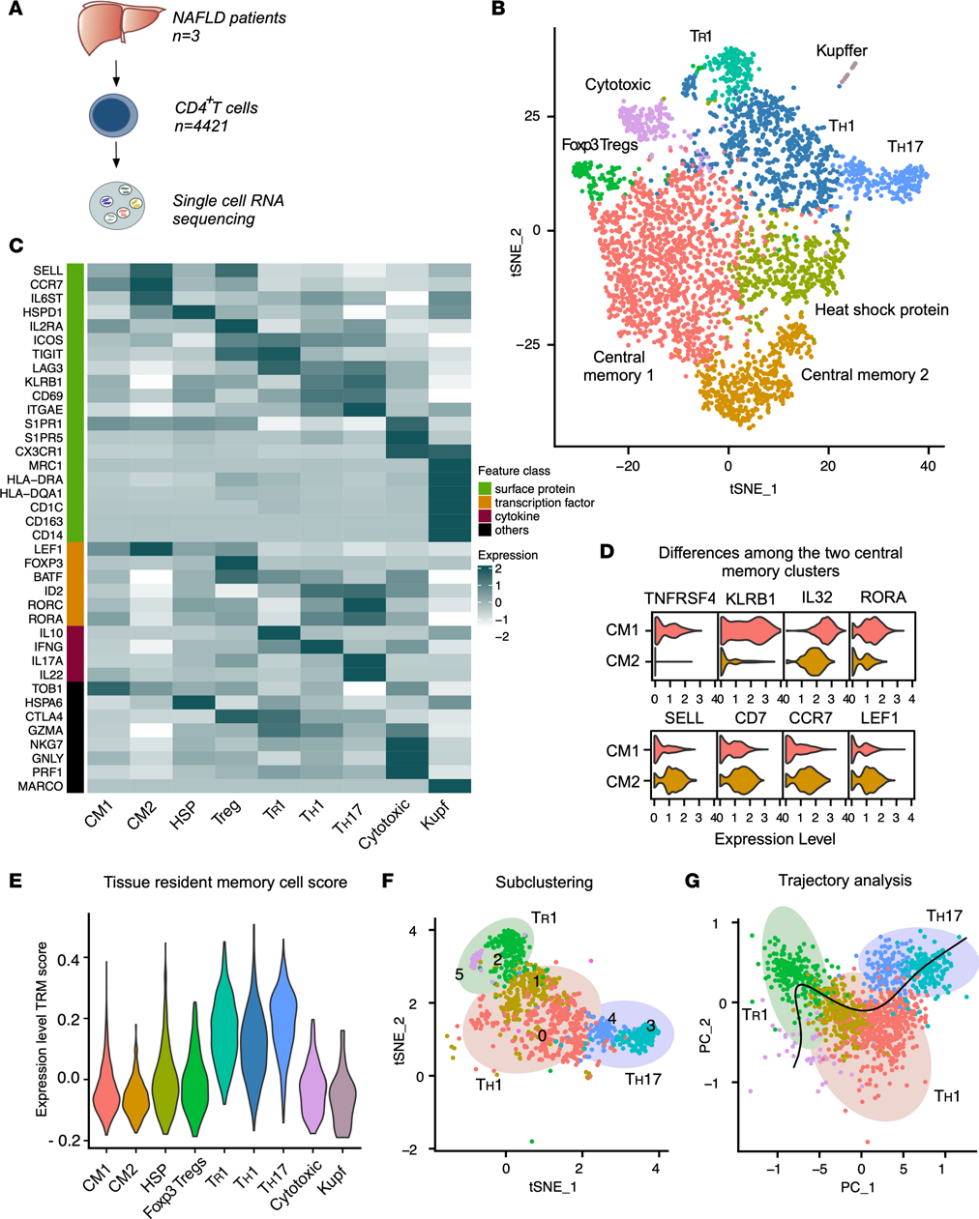
scRNA-seq of CD4^+^ T cells found in the liver of NAFLD patients. (**A**) Schematic of experimental setup: FACS-isolated CD45RA^–^CD4^+^ T cells (CD4^+^ T cells) from liver tissue of NAFLD patients were processed for scRNA-seq. (**B**) t-SNE plot of the unsupervised clustering of CD4^+^ T cells. (**C**) Heatmap of CD4^+^ T cell clusters displaying key signature genes to annotate the different clusters. CM1 and CM2, central memory populations 1 and 2. (**D**) Violin plots comparing the most differentially expressed genes between the central memory clusters. (**E**) Expression of the tissue-resident memory T cell (Trm) gene signature score of the indicated clusters of human CD4^+^ T cells. (**F**) t-SNE plot of the subclustering of Tr1, Th1, and Th17 clusters. (**G**) PCA plot of slingshot pseudotime developmental trajectory analysis.

To describe CD4^+^ T cells infiltrating the liver of NAFLD patients in detail, we performed scRNA-seq (*n* = 4421 cells) on FACS-isolated memory CD45RA^–^CD4^+^ T cells isolated from 3 NAFLD patients (Figure 1A, Supplemental Figure 1A, and Supplemental Table 1; supplemental material available online with this article; https://doi.org/10.1172/jci.insight.153831DS1). An unsupervised analysis revealed 8 distinct T cell clusters, which were annotated by examining the most differentially expressed genes and comparing our data set to standard markers used in flow cytometry and already existing sequencing data of cells in the liver (15–19) (Figure 1B). We found populations of CD4^+^ T cells with either a Th1 (*IFNG*) or a Th17 (*IL17A*, *RORA*) polarization state. Of note, CD4^+^ T cells with a Th17 polarization state expressed both RORA and RORC, as previously reported (20). We also found cells with transcriptomes resembling those of antiinflammatory type 1 Treg (Tr1: *IL10*, *TIGIT*, *LAG-3*, *CTLA4*) and Foxp3^+^ Treg (*FOXP3*, *CTLA4*, *IL2RA*) cells. Central memory cells (*CCR7*, *SELL*), heat shock protein–positive (HSP)^+^ cells, and cytotoxic (*GNLY*, *PRF1*) cells were also identified. Finally, we found a small contamination of Kupffer cells (*CD163*, *CD14*) (Figure 1, B and C).

Regarding the central memory cells, we found 2 distinct cell populations. Central memory cell population 2 showed a less active cell signature, expressing high levels of *SELL*, *CD7*, *CCR7*, and *LEF1* (21) compared with population 1, which was characterized by high expression of *TNFRSF4*, *KLRB1*, *IL32*, and *RORA* (Figure 1D), suggesting active memory cells with possible proinflammatory activity. We did not identify distinct cell clusters resembling Th2, Th9, and T follicular helper cell (Tfh) polarization states. By performing a supervised analysis looking for key markers, we found that few cells expressed *IL4*, some *BCL6* but simultaneously not *CXCR5*, and none *IL9*. *GATA3* was expressed at moderate levels on virtually all the cells (Supplemental Figure 1B). The low resolution of the scRNA-seq could explain these findings and these data do not exclude the presence of Th2, Th9, and Tfh polarization states in the liver of NAFLD patients.

Besides circulating central memory and effector memory cells, tissue-resident memory (Trm) cells play a crucial role in orchestrating both protective and pathological tissue-specific immune responses (22, 23). Therefore, we wondered which of the above-mentioned populations of CD4^+^ T cells display a Trm transcriptomic signature. By using a literature-curated Trm signature score (24) that we recently validated (25), we observed that the Th cell clusters, namely Th1, Th17, and Tr1, were enriched for the Trm gene signature (Figure 1E). This suggests that cells with a Trm-like signature comprise a heterogeneous group of Th cell subsets, probably reflecting their effector precursors.

It has been shown that a distinct polarized CD4^+^ T cell subset, such as Th17, might acquire the phenotype of another subset such as Th1 or Tr1 (26–28). To further investigate this in our liver CD4^+^ T cell subsets, we first performed a subclustering analysis of Th1, Tr1, and Th17 cells. We found 6 different subclusters reflecting different cell states (Figure 1F and Supplemental Figure 1C) with combined Th1, Tr1, and Th17 features and partial overlap between the different states. Next, we inferred differentiation cell states by performing 2 distinct trajectory analyses (i.e., Slingshot and Monocle 3) (Figure 1G and Supplemental Figure 1, D and E). Both analyses revealed a potential cell trajectory connecting Th17 and Th1 and further Tr1 cell states. Hence, we postulate that some of these Th cells, characterized by a Trm gene signature, might represent a unique cellular spectrum composed of different cellular states.

Overall, our analysis reveals the landscape of the CD4^+^ T cell populations in the liver of NAFLD patients, including 1 population of cytotoxic effector memory T cells, 2 distinct central memory populations, and Trm-like cells characterized by a continuum of different cell states, namely Th1, Th17, and Tr1 cells.

### Characterization of 8 distinct subsets of liver-infiltrating CD4^+^ T cells in NASH patients.

**Figure 2:**
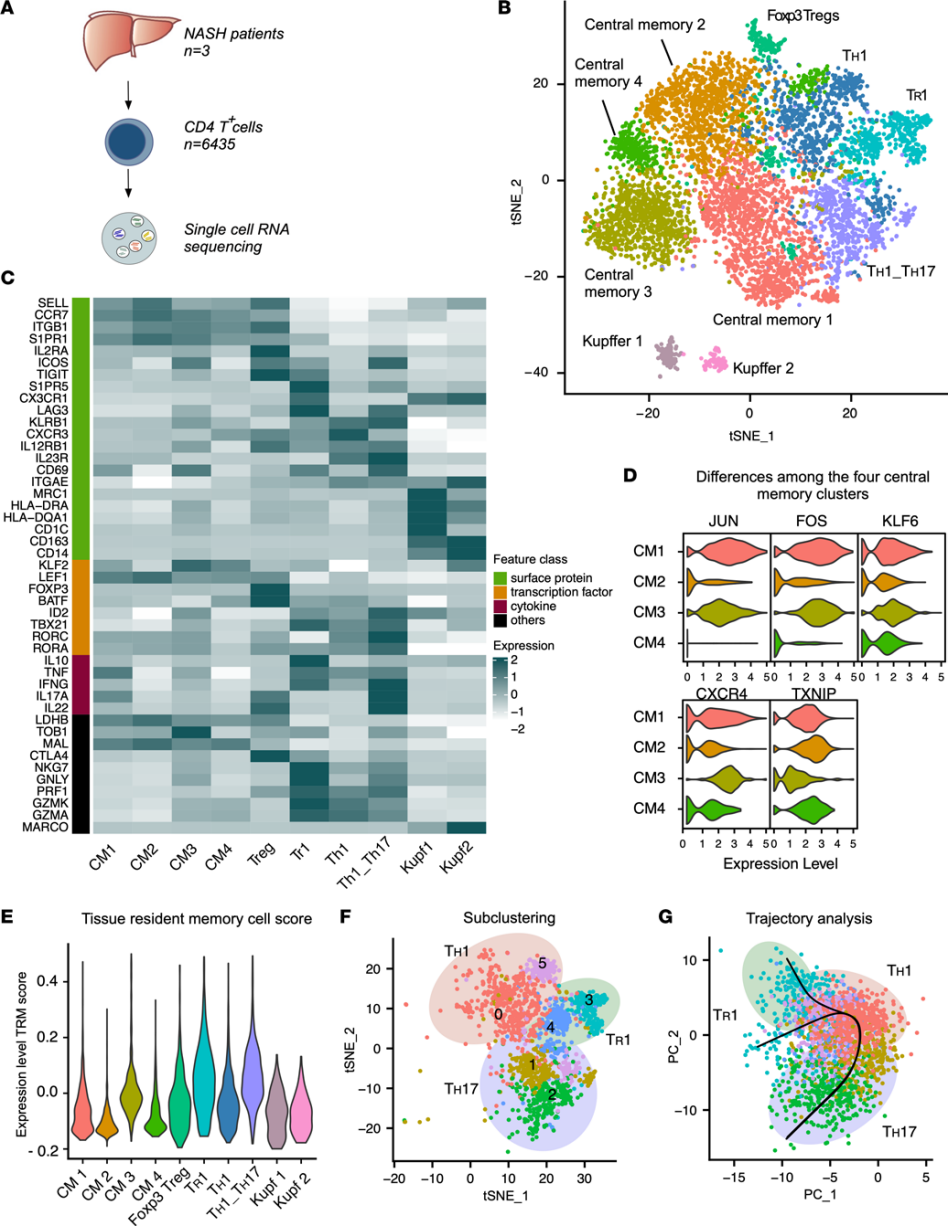
scRNA-seq of CD4^+^ T cells found in the liver of NASH patients. (**A**) Schematic of experimental setup: FACS-isolated CD45RA^–^CD4^+^ T cells (CD4^+^ T cells) from liver tissue of NASH patients were processed for scRNA-seq. (**B**) t-SNE plot of the unsupervised clustering of CD4^+^ T cells. (**C**) Heatmap of CD4^+^ T cell clusters displaying key signature genes to annotate the different clusters. CM1 and CM2, central memory populations 1 and 2. (**D**) Violin plots comparing the most differentially expressed genes between the central memory clusters. (**E**) Expression of the tissue-resident memory T cell (Trm) gene signature score of the indicated clusters of human CD4^+^ T cells. (**F**) t-SNE plot of the subclustering of Tr1, Th1, and Th17 clusters. (**G**) PCA plot of slingshot pseudotime developmental trajectory analysis.

Next, we wondered whether the above-mentioned populations of liver CD4^+^ T cells were specific to NAFLD patients or also present in NASH. Therefore, we performed scRNA-seq (*n* = 6435) on CD45RA^−^CD4^+^ T cells isolated by FACS from the liver of 3 NASH patients (Figure 2A and Supplemental Table 1) and identified 8 different T cell clusters.

Among these cell clusters, we found CD4^+^ T cells with Th1, Th1/Th17, and Tr1 polarization states and Foxp3^+^ Treg cells (Figure 2, B and C). The cell clusters were annotated as described above for the NAFLD data set.

In addition, our analysis revealed 4 different clusters of central memory cells, all expressing SELL and CCR7. The central memory cell populations 1 and 3 showed higher expression of JUN and FOS, and in addition, population 3 showed the lowest expression of TXNIP, suggesting that these 4 populations might reflect different activation states of central memory cells (Figure 2D). In accordance with the NAFLD map, we did not identify specific clusters corresponding to Th2, Th9, or Tfh subsets (Supplemental Figure 2A). Using the Trm gene signature score, we observed that CD4^+^ T cells with Th1, Th1/Th17, and Tr1 polarization states had the highest relative expression score compared with the other cell cluster (Figure 2E).

To further characterize potential transitional cell states, we subclustered the Th1, Th1/Th17, and Tr1 clusters and identified 6 different subpopulations (Figure 2F and Supplemental Figure 2B) and again, found partial overlap between the different states. However, in contrast to the NAFLD map, both pseudotime analyses showed more chaotic paths with different potential cellular endpoints, including the potentially cytotoxic Tr1 subcluster 3 (*IL10*, *TIGIT*, *GZMH*, *GZMB*, *PRF1*, *GZMK*) (Figure 2G and Supplemental Figure 2, C and D). These chaotic paths suggest that a less delineated T effector cell trajectory is present in NASH compared with NAFLD patients.

### Differences revealed between liver CD4^+^ T cells in NAFLD and NASH patients.

**Figure 3:**
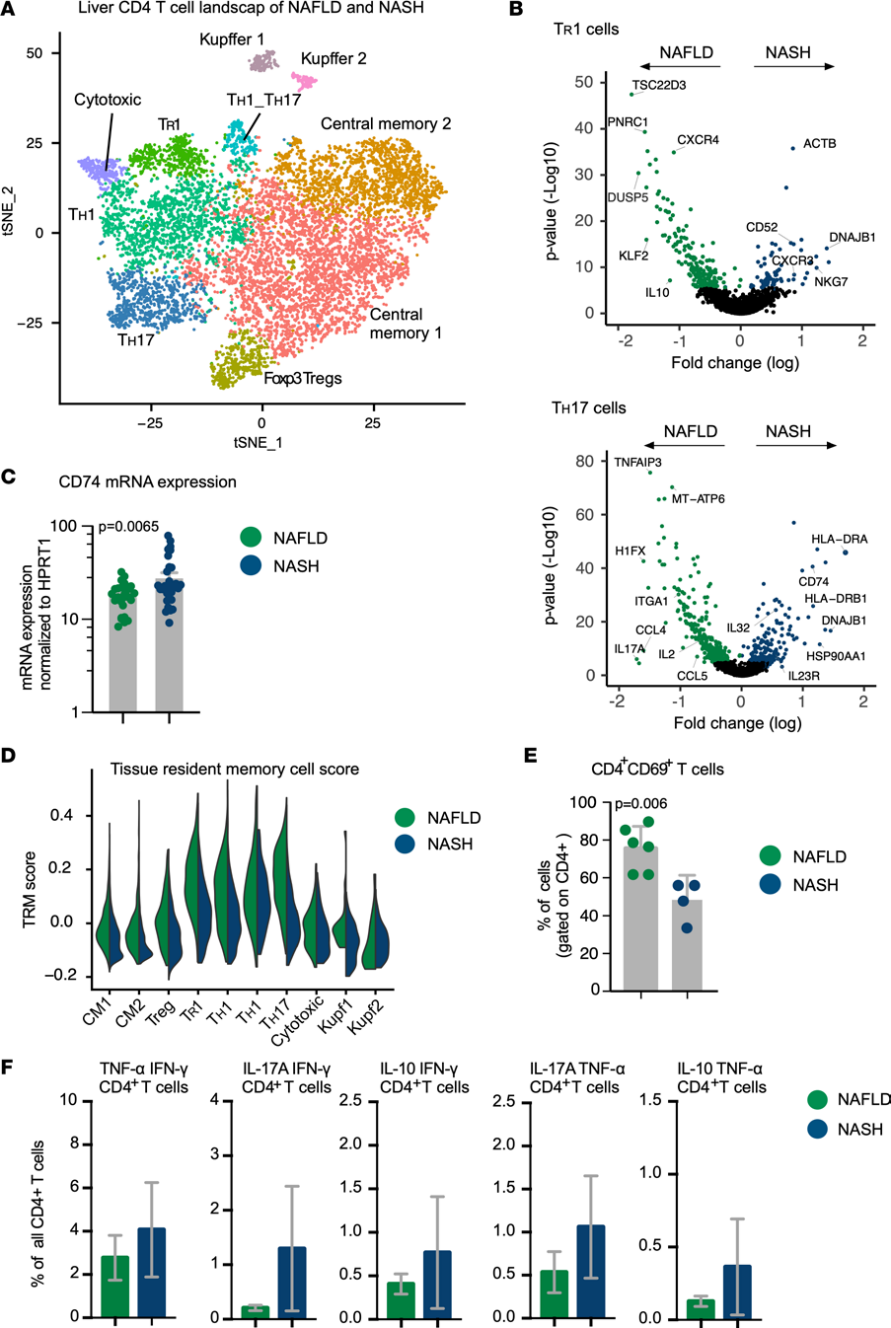
Differences between CD4^+^ T cells in the livers of NAFLD and NASH patients. (**A**) t-SNE plot of the unsupervised clustering of CD45RA^–^CD4^+^ T cells (CD4^+^ T cells) found in the liver of NASH and NAFLD patients. This is a combined analysis of the same data set shown in Figures 1 and 2. (**B**) Volcano plots showing the differential gene expression profiles between NAFLD and NASH of liver Tr1 and Th17 cells. (**C**) Real-time PCR of *CD74* in total liver tissues from NAFLD (*n* = 25) and NASH (*n* = 32) patients. Each dot represents 1 patient. Data are presented as mean ± SEM. *P* value was determined by Mann-Whitney *U* test. (**D**) Expression of the tissue-resident memory T cell (Trm) gene signature scores of the indicated clusters of human CD4^+^ T cells in NAFLD and NASH. CM1 and CM2, central memory populations 1 and 2. (**E**) Frequencies of CD69^+^ cells within CD4^+^ T cells obtained using FACS. Data are presented as mean ± SEM. Each dot represents 1 patient. *P* value was determined by Mann-Whitney *U* test. (**F**) Frequencies of the indicated multicytokine-expressing CD4^+^ T cells based on the scRNA-seq data set (NAFLD *n* = 3, NASH *n* = 3). Data are presented as mean ± SEM.

To further investigate differences between NAFLD and NASH, we decided to integrate in 1 analysis the 2 CD4^+^ T cell data sets. We found that each cluster contained cells from NASH and NAFLD patients and nearly all clusters were equally represented by all specimens and proportionally distributed across patients (Figure 3A and Supplemental Figure 3, A and B).

Next, we wondered whether the transcriptional profiles of these cell clusters were different between NAFLD and NASH. Differential gene expression analysis of IL-10–producing Foxp3^–^CD4^+^ T cells — referred to so far as Tr1 cells — revealed that *IL10* was more highly expressed in the cells from the NAFLD patients, while the chemokine receptor gene *CXCR3* was significantly overexpressed in IL-10–producing Foxp3^–^CD4^+^ T cells derived from NASH patients (Figure 3B). Of note, CXCR3 was shown to be associated with overproduction of cytokines (29). In line with that, our recently published data show that IL-10–producing Foxp3^–^CD4^+^ T cells are a functionally heterogeneous population of cells with both antiinflammatory function and potential pathogenic activity (16). Therefore, we created a Tr1 score based on our previously published data (16) and found that the IL-10–producing Foxp3^–^CD4^+^ T cells in NASH had a lower Tr1 score compared with those in the liver of NAFLD patients (Supplemental Figure 3C). This suggests an enrichment of non-Tr1 IL-10–producing Foxp3^–^CD4^+^ T cells in NASH.

Next, we focused on the CD4^+^ T cells with a Th17 cell polarization state, since these cells have been suggested to play a role in NASH development (6, 30). We therefore performed a differential gene expression analysis of these cells between NAFLD and NASH. *IL23R*, *IL32*, and *CD74* were upregulated in the cells isolated from NASH patients compared with those from NAFLD, suggesting a possible proinflammatory Th17 cell state in NASH patients (Figure 3B). Moreover, when the differential gene expression analysis was also performed on other cell clusters, including Th1 and Foxp3^+^ Treg cells, we observed that *CD74* was more highly expressed in the cells isolated from NASH patients compared with NAFLD patients (Supplemental Figure 3D and Supplemental Table 2). To validate this finding, we extended our analysis to 25 NAFLD and 32 NASH patients by testing *CD74* expression by qPCR analysis. We found that the expression of *CD74* overall was significantly higher in NASH patients (Figure 3C).

Next, since Th1, Th17, and Th1/Th17 polarization cell states displayed a Trm gene signature in both NAFLD and NASH, we compared their score across the 2 liver diseases. We found a higher expression of the Trm transcriptomic signature in the liver cells isolated from the NAFLD patients (Figure 3D).

In order to validate these findings, we performed flow cytometric analysis and confirmed the enrichment of CD69^+^CD4^+^ T cells in NAFLD patients (Figure 3E and Supplemental Figure 3E). CD69 is one of the most utilized markers of Trm cells (31). Despite the limitation of being influenced by cell stimulation, together with our scRNA-seq analysis, these data suggest that a higher concentration of cells with a Trm-like phenotype are present in the liver of NAFLD patients.

In addition to this, we found that CD4^+^ T cells with Th1 and Th17 polarization states isolated from the liver of the NASH patients expressed some of the intestine-related chemokine and integrin genes such as *CCR6*, *CCR9*, and *ITGA4* (Supplemental Figure 3F). Whether this indicates a liver-intestine migration of the T cells remains to be determined.

To further confirm our findings, we performed an integrated analysis using a published scRNA-seq liver data set that includes heathy liver–derived T cells (19). After integrating our data set with the published data set, we first recognized the same clusters of CD4^+^ T cells, and secondly, when we compared the Trm score across healthy, NAFLD, and NASH conditions we confirmed that CD4^+^ T cells with Th1 and Th17 polarization states isolated from NASH patients had the lowest score (Supplemental Figure 4, A–D).

Finally, since our differential gene expression analysis of Tr1 and Th17 cells revealed genes connected with the activation of T cells and an increase in cytokine production, such as *CXCR3*, *IL23R*, and *CD74*, we analyzed our single-cell sequencing data set for the frequency of cytokine-producing CD4^+^ T cells. Despite the fact that the low number of patients did not allow us to perform a statistical test, it appeared that a higher percentage of cells coexpressing a combination of *TNF*, *IFNG*, *IL17A*, and *IL10* (Figure 3F) was present in NASH compared with NAFLD.

In conclusion, we found that NASH-derived IL-10–producing Foxp3^–^CD4^+^ T cells and CD4^+^ T cells with a Th17 cell polarization state were characterized by a nonregulatory transcriptional profile and a more pathogenic gene signature, respectively. In addition, CD4^+^ T cells found in the liver of NASH patients appeared to have a low Trm-like transcriptional profile and expressed genes encoding some chemokines usually expressed by intestinal T cells. Finally, all the different CD4^+^ T cell clusters found in the NASH patients seemed to have an overall proinflammatory transcriptomic signature characterized, for example, by the coexpression of many proinflammatory cytokines.

### The livers of NASH patients were characterized by an enrichment of CD4^+^ T cells with a multicytokine profile.

**Figure 4:**
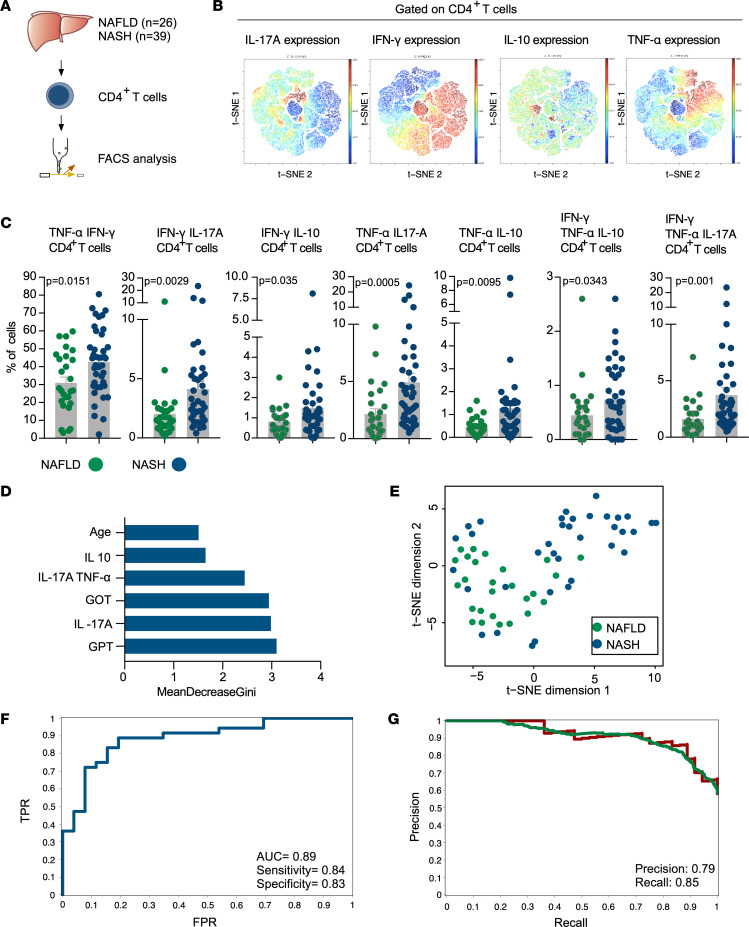
Liver CD4^+^ T cells in NASH had a distinct cytokine profile compared with NAFLD. (**A**) Schematic of experimental setup: CD45RA^–^CD4^+^ T cells (CD4^+^ T cells) were freshly isolated from liver tissue of NAFLD and NASH patients, restimulated in vitro, and analyzed by FACS for multiple cytokines (i.e., IFN-γ, TNF-α, IL-17A, IL-22, IL-22BP, IL-4, and IL-10) and the transcriptional factor FoxP3. (**B**) Representative t-SNE analysis of liver-infiltrating CD4^+^ T cells of 5 NASH patients. (**C**) Frequencies of the indicated multicytokine-producing CD4^+^ T cells based on FACS analysis. Each dot represents 1 patient. Data are presented as mean ± SEM. *P* values were determined by Mann-Whitney *U* test. (**D**) Top 6 features to predict NAFLD or NASH based on random forest mean decrease in Gini score (GOT: glutamic oxaloacetic transaminase; GPT: glutamic-pyruvic transaminase). (**E**) t-SNE visualization plot of NAFLD and NASH patients based on the previously indicated top 6 features of prediction. Each dot represents 1 patient. (**F**) Receiver-operating characteristic curve showing true- and false-positive rates (TPR and FPR, respectively) for the discrimination between NAFLD and NASH prediction based on the top 6 features of prediction. (**G**) Precision and recall plot for prediction based on indicated top 6 features of prediction.

To validate the observation regarding the increased frequency of liver CD4^+^ T cells able to coproduce multiple cytokines in NASH patients, we performed intracellular cytokine FACS staining on CD4^+^ T cells freshly isolated from the liver of 26 NAFLD and 39 NASH patients (Figure 4A, Supplemental Figure 5A, and Supplemental Table 3). Considering that many cytokine combinations can possibly occur, we performed a viSNE analysis to identify which of them are indeed present in the liver of at least either NAFLD or NASH patients (Figure 4B). We found that different populations of multicytokine-producing CD4^+^ T cells are significantly enriched in the liver of NASH compared with NAFLD patients, namely cells coproducing TNF-α/IFN-γ, IFN-γ/IL-17A, IFN-γ/IL-10, TNF-α/IL-17A, TNF-α/IL-10, TNF-α/IFN-γ/IL-10, and TNF-α/IFN-γ/IL-17A (Figure 4C). The expression of IL-22 was low and mainly expressed by those cells expressing IL-17A. Finally, we found low frequencies of CD4^+^ T cells expressing IL-22 binding protein (IL-22BP) and a consistent frequency of CD4^+^ T cells expressing IL-4 (i.e., Th2 polarization state), but we did not see any difference between NAFLD and NASH patients (Supplemental Figure 5, A and B).

Then, we wanted to further test the relevance of the above-identified multicytokine-producing cells to distinguish between NASH and NAFLD using a random forest classifier. As reference, we used clinical parameters, such as transaminase levels, since they are known to discriminate between NASH and NAFLD, but still with a low sensitivity and specificity. By random forest machine learning, we identified the most important features to discriminate between NAFLD and NASH, which include clinical parameters as well as IL-10–, IL-17A–, and TNF-α–producing CD4^+^ T cells and IL-17A/TNF-α–coproducing CD4^+^ T cells (Figure 4D). Visualization by t-distributed stochastic neighbor embedding (t-SNE) analysis indeed showed that NAFLD and NASH patients segregated from each other, based on these features (Figure 4E). In line with this, using the selected features, a classification performance of 89% was obtained that distinguished between NAFLD and NASH patients (AUC = 0.89, sensitivity = 0.84, specificity = 0.83) (Figure 4, F and G). As a control, we used the clinical parameters alone (sex, age, BMI, and transaminase levels) or cytokine-producing cells alone, and both showed less accuracy (AUC = 0.79 and AUC = 0.69; Supplemental Figure 5, C and D) than when combined.

Taken together, our results show that populations of multicytokine-producing CD4^+^ T cells were significantly enriched in the liver of the NASH patients and we were able to distinguish between NAFLD and NASH patients in combination with clinical parameters.

### CD4^+^ T cells with a Th17 polarization state are enriched in the fibrotic livers of NASH patients.

**Figure 5:**
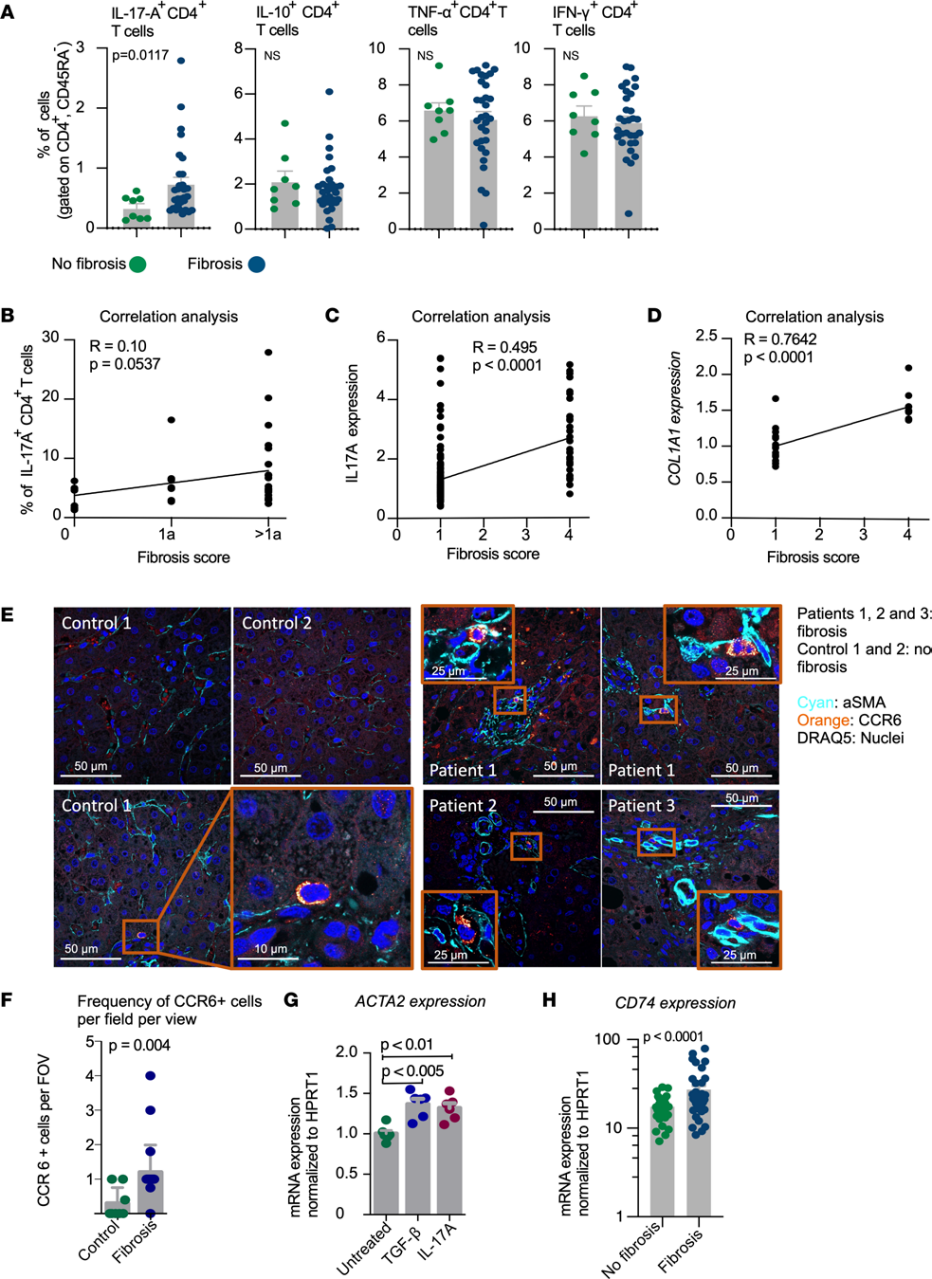
IL-17A–producing CD4^+^ T cells were enriched in the liver of NASH patients with fibrosis. (**A**) Comparison of the FACS analysis of the frequencies of the indicated multicytokine-producing CD4^+^ T cells isolated from the NASH patients with and without fibrosis (same cohort as in Figure 4). Each dot represents 1 patient. Data are presented as mean ± SEM. *P* values were determined by Mann-Whitney *U* test. NS, not significant (*P* > 0.05). (**B**–**D**) Correlations between fibrosis score and (**B**) frequencies of liver IL-17A–producing CD4^+^ T cells obtained by FACS, (**C**) *IL17A* expression obtained form the scRNA-seq data set, and (**D**) *COL1A1* expression obtained from real-time PCR of total liver tissue of NASH patients undergoing bariatric surgery. The *COL1A1* expression is normalized to *HPRT* expression. *P* values were calculated with Pearson’s correlation coefficient. (**E**) Immunofluorescent staining of the livers of patients with fibrosis (patients 1, 2, and 3) and without fibrosis (control 1 and 2). αSMA in cyan, CCR6 in orange, and DRAQ5 in far red for nuclei. (**F**) Frequency of CCR6^+^ cells per field of view (FOV). Each dot represents the number of CCR6^+^ cells per FOV. Data are presented as mean ± SEM. *P* value was determined by unpaired *t* test with Welch’s correction. (**G**) Real-time PCR of *ACTA2* in fibroblasts either left untreated or stimulated in vitro with TGF-β (10 ng/mL) and IL-17A (150 ng/mL) for 24 hours. Data are a pool of 2 independent experiments and each dot represents the *ACTA2* expression obtained from each culture well. Data are presented as mean ± SEM. *P* values were determined by unpaired 1-way ANOVA test. (**H**) Real-time PCR of *CD74* in liver tissues of NASH patients with and without fibrosis. The *CD74* expression is normalized to *HPRT* expression. Each dot represents 1 patient. Data are presented as mean ± SEM. *P* value was determined by Mann-Whitney *U* test.

We wondered whether the frequency of the liver multicytokine-producing CD4^+^ T cells correlated with the fibrosis score in NASH patients. Among the different multicytokine-producing CD4^+^ T cells, we discovered that CD4^+^ T cells producing mainly IL-17A, but not exclusively — i.e., CD4^+^ T cells with a Th17 polarization state — were significantly enriched in the liver of NASH patients with fibrosis compared with no fibrosis (Figure 5A). Furthermore, there was a correlation between the frequency of these CD4^+^ T cells and the fibrosis score (Figure 5B).

These findings were confirmed using our scRNA-seq data set, showing that the expression of *IL17A* correlated positively with the fibrosis score in NASH patients (Figure 5C). Of note, the accuracy of the fibrosis score was confirmed by a significant positive correlation with the expression of the profibrotic gene *COL1A1* (Figure 5D).

To further confirm our findings in a different cohort of patients, we used a publicly available human NASH bulk RNA-seq data set (32), and found a significant correlation between the expression of *IL17A* and *COL1A1* (Supplemental Figure 5E).

We next wondered whether the liver IL-17A–producing CD4^+^ T cells were in close proximity to liver cells that might be responsible for fibrosis, such as hepatic stellate cells and fibroblasts. We therefore performed immunofluorescent staining using CCR6 as marker for IL-17A–producing CD4^+^ T cells, and α-smooth muscle actin (αSMA) as marker for the cells potentially responsible for liver fibrosis. In the 2 nonfibrotic control livers, we observed that CCR6^+^ cells were very rare. In contrast, in the 4 patients with fibrosis, CCR6^+^ cells were significantly more frequent, and in most cases, they were located near αSMA^+^ structures (Figure 5, E and F).

Finally, we performed a proof-of-concept in vitro experiment to test whether the cytokine IL-17A can activate fibroblasts. As a positive control we used TGF-β, a key activator of fibroblasts (33). We found that IL-17A might be able to activate fibroblasts, as measured by the increased gene expression of the activation marker *ACTA2* as compared with the untreated fibroblasts (Figure 5G).

Next, since we found the expression of *CD74* to be significantly higher within the cluster of *IL17A*-expressing CD4^+^ T cells of the NASH patients (Figure 3B), we wondered whether *CD74* is also enriched in patients with fibrosis. In a different cohort of patients, analysis of *CD74* expression by qPCR did indeed reveal a significantly higher expression in NASH patients with fibrosis compared with those without (Figure 5H).

Taken together, our results show that multicytokine-producing CD4^+^ T cells, mainly characterized by the expression of IL-17A, are particularly enriched in the liver of those NASH patients with fibrosis compared with those without fibrosis. The role of CD74 in this context remains to be further studied.

### Characterization of the microbiota in the small intestines and livers of NAFLD and NASH patients.

**Figure 6:**
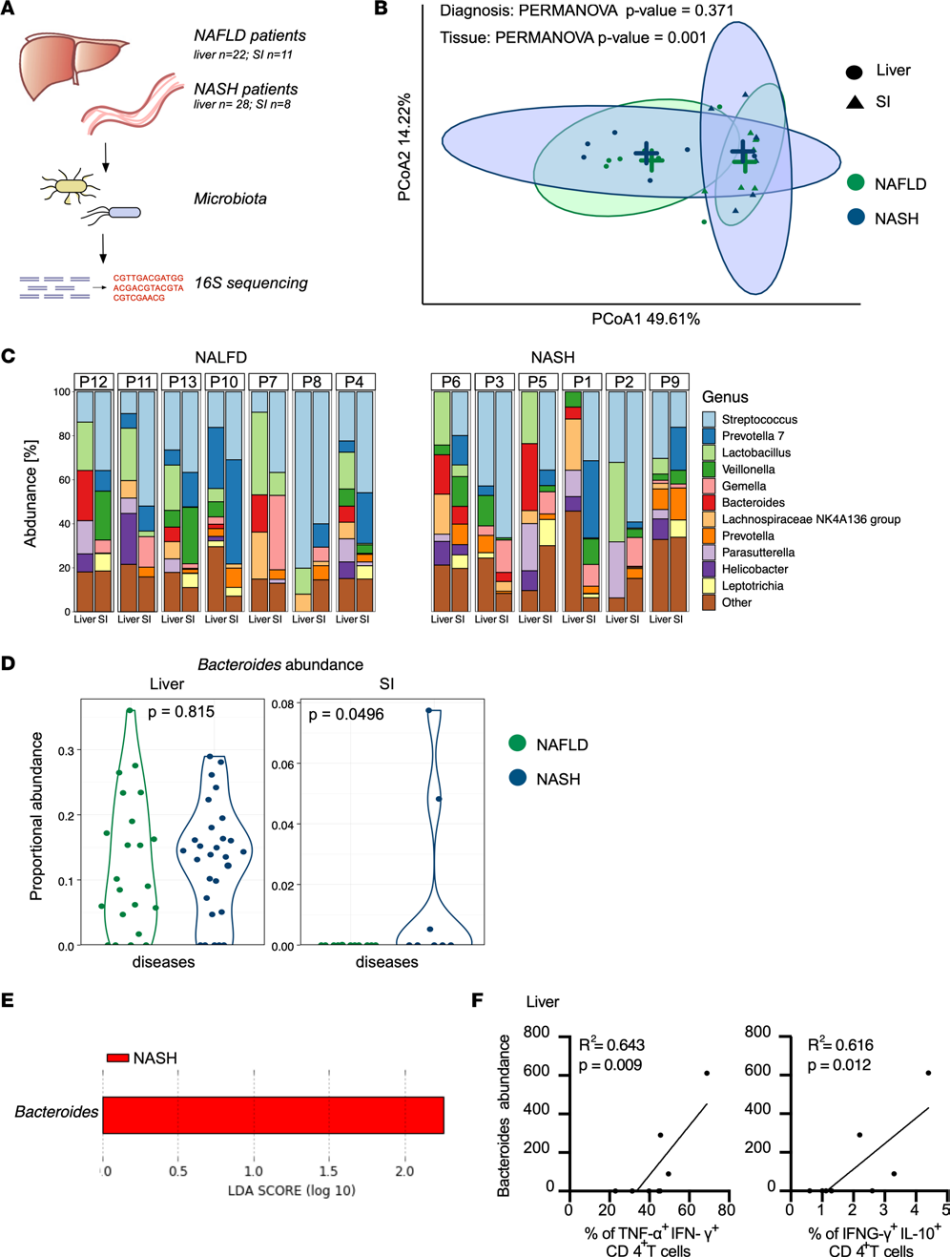
Characterization of the microbiota in the small intestines and livers of NAFLD and NASH patients. (**A**) Schematic of experimental setup: Microbiota isolated from liver and small intestine (SI) was subjected to *16s* rRNA sequencing. (**B**) PCoA plot showing the differences of microbial β-diversity between NAFLD and NASH, in the livers and SI. Each dot represents 1 patient. *P* value was calculated with the PERMANOVA test. (**C**) Differential abundance of bacteria in NAFLD and NASH patients (P) in liver and SI. (**D**) Proportional abundance of *Bacterioides* in liver and SI of NAFLD and NASH patients. *P* values were determined by the fitFeatureModel from the R package metagenomeSeq. (**E**) LEfSe analysis of the bacterial species enriched in the SI of 11 NAFLD and 8 NASH patients. LDA, logarithmic discriminant analysis. (**F**) Correlation between *Bacteroides* abundance and frequency of the indicated T cell populations in the liver. Each dot represents 1 patient. *P* values were calculated with Pearson’s correlation coefficient.

Considering the known link between CD4^+^ T cell function and intestinal microbiota (9), our finding regarding the enrichment of multicytokine-producing CD4^+^ T cells in NASH patients led us to argue in favor of a possible liver-gut axis as a reason of the hyperactivation of the liver T cells. To support this, instead of analyzing the fecal microbiome, we directly tested (*16s* rDNA sequencing) the composition of the tissue-specific microbiome of both livers (*n* = 50) and small intestines (*n* = 19) of the NAFLD and NASH patients (Figure 6A).

We first confirmed the presence of bacteria in the liver of both NAFLD and NASH patients (34) (Supplemental Figure 6A). Second, we examined differences in the microbiome structure between the liver and small intestine across NAFLD and NASH patients by principal coordinate analysis (PCoA) of Bray-Curtis distance (Supplemental Figure 6A). The analysis revealed similar microbial communities within NASH and NAFLD patients. Furthermore, the within-sample diversity (α-diversity, Shannon’s diversity) did not differ between different disease conditions (Supplemental Figure 6B). To adjust for interpatient variability, we next analyzed paired liver and small intestine samples, and observed that the microbial composition between NAFLD and NASH patients did not differ (Figure 6B).

Next, we explored the community structure at the genus level and found that some bacteria were present in both the small intestine and liver of patients (Figure 6C). In addition we observed that *Bacteroides* were significantly enriched in the small intestine of the NASH patients (Figure 6, D and E). *Bacteroides* were also present in the liver tissue, but there were no significant differences in genus abundances between NAFLD and NASH in our cohort of patients (Figure 6D).

Finally, we wondered whether the relative amount of *Bacteroides* correlated with an enrichment in the multicytokine-producing CD4^+^ T cells.

First, we performed intracellular cytokine FACS staining on CD4^+^ T cells from the small intestine of 15 NAFLD and 10 NASH patients, as previously performed for the liver CD4^+^ T cells. Despite the fact that the data did not show any statistical significance, we observed that there was an enrichment of cells coexpressing cytokines in the intestine of NASH patients compared with NAFLD (Supplemental Figure 6, C and D). Second, we tested for a possible correlation between multicytokine-producing CD4^+^ T cells in the liver and small intestine and *Bacteroides* abundance in NASH patients. Despite the low number of samples analyzed, we observed a significant positive correlation between the frequency of liver CD4^+^ T cells coproducing TNF-α/IFN-γ and IFN-γ/IL-10 and liver *Bacteroides* abundance in 3 patients (Figure 6F), and only by trend between intestinal CD4^+^ T cells coproducing TNF-α/IFN-γ and IFN-γ/IL-10 and intestinal *Bacteroides* abundance (Supplemental Figure 6E).

In short, these data showed the presence of some of the intestinal bacteria in the liver of both NAFLD and NASH patients, but the composition of the liver microbiota did not differ between the 2 disease stages. In contrast, *Bacteroides* appeared to be enriched in the mucosal layer of the small intestine of NASH patients. The analysis of the *Bacteroides* is limited by the low number of patients analyzed (*n* = 8); however, the results are in line with what has been previously published by sequencing the stool of NASH patients (34). Finally, a possible correlation seems to be present between the frequency of multicytokine-producing liver CD4^+^ T cells and *Bacteroides*. Whether these data support a liver-gut axis and could be the reason for the accumulation multicytokine-producing liver CD4^+^ T cells can only be hypothesized.

## Discussion

By providing an unsupervised analysis, our study shows for the first time to our knowledge that the progressive inflammatory development behind the transition from NAFLD to NASH is not associated with only classical CD4^+^ Th cell subsets, as previously suggested (6, 30, 35, 36), but rather with a larger spectrum of different subsets, including multicytokine-producing cells that might represent in-between developmental stages.

Using fate-mapping mouse models, we have previously shown that Th17 cells can either have a proinflammatory fate, participating in a pathological immune response, or an antiinflammatory fate, contributing to the resolution of the immune response (27, 28). Here, by combining an unsupervised transcriptional profiling and multiparameter flow cytometric analyses, we describe a dynamic interconnection among CD4^+^ T cells with Th1, Th17, and Tr1 cell states. Moreover, we show that there is a more chaotic developmental path with different pathogenic endpoints and an increase in multicytokine-producing CD4^+^ T cells in NASH patients that could be one of the causes of the chronic liver inflammation typical of these patients. However, since our findings are based on a descriptive set of analyses, further experiments using, for example, fate-mapping mouse models are needed to confirm the possible developmental interconnection among liver CD4^+^ T cells.

Within the CD4^+^ T cells with Th17, Th1, and Tr1 polarization states and Foxp3^+^ Treg cells we found *CD74* to be enriched in NASH patients and in those characterized by fibrosis. CD74 is usually not known to be expressed on T cells, but it was previously reported to regulate proliferation, survival, and secretion of inflammatory cytokines, after activation by MIF (37–39). In a mouse model of chronic liver injury, CD74-deficient mice were protected from liver fibrosis (39). Therefore, CD74 could be a potential novel target to disrupt NASH progression and development of fibrosis, by using an anti-CD74 antibody such as milatuzumab (also known as hLL1), for example (40, 41). Investigating CD4^+^ T cell–specific CD74 ablation would determine whether CD74 on T cells is involved in NASH and fibrosis.

The role of Trm cells in the development of NASH is largely unknown. We showed that the percentage of cells with a Trm gene signature and phenotype is higher in NAFLD than in NASH patients. This finding was confirmed using a different cohort of patients from a previously published data set (19). Considering all these data together, a possible working hypothesis is that CD4^+^ T cells found in the liver of NASH patients have a more circulatory nature compared with those found in the NAFLD livers. In addition, considering the expression of some chemokine receptors typical of intestine-derived T cells, we speculate that the multicytokine-producing liver CD4^+^ T cells had previously been activated in mucosa-associated lymphoid tissue or in the intestine and then migrate through the liver.

In line with this, emerging evidence indicates that intestinal dysbiosis has a significant role in the pathogenesis of human liver diseases. Dysbiosis was shown to associate with intestinal permeability (42) and consequently lead to translocation of microorganisms and microorganism-derived molecules into the liver (14, 43). Using *16s* rRNA gene sequencing of fecal samples, Boursier et al. found that the bacterial genera *Bacteroides* and *Ruminococcus* were substantially higher, and *Prevotella* was lower in patients with NASH compared with those without NASH (12). Moreover, Sookian et al. showed that the liver tissue of NAFLD patients contains a diverse repertoire of bacterial DNA that differs between morbidly obese and non–morbidly obese NAFLD patients (14). Here, we investigated for the first time to our knowledge the tissue-specific microbiome of both the liver and small intestine within NASH and NALFD morbidly obese patients. Our data confirm the presence of intestinal microbiota in the liver of both NAFLD and NASH patients. However, within the liver tissue of morbidly obese NAFLD and NASH patients, the microbial communities were similar. Because obesity was shown to be an important driver of liver microbial DNA composition (14), this might explain the difference between — but not within — morbidly obese and non–morbidly obese patients. In contrast, investigating the microbial composition of the intestinal mucosal layer, our data suggest that *Bacteroides* might be enriched in the small intestine of some morbidly obese NASH patients. This is in line with studies showing an increase in *Bacteroides* abundance in stool samples of NASH patients (12). Interestingly, some of the patients with elevated levels of *Bacteroides* in the liver also displayed elevated percentages of multicytokine-producing CD4^+^ T cells, above median levels. We here speculate that in NASH patients, the abundance of some intestinal bacteria (e.g., *Bacteroides*) might predispose T cells to a possible dysfunctional activity. Interestingly, upon potential migration to the liver, these T cells find some of the intestinal bacteria, which in combination with a fat-enriched environment might unleash the predisposition of T cells to secrete many cytokines simultaneously participating in the development of NASH.

Finally, we report the persistence of CD4^+^ T cells with a Th17 polarization state in the development of fibrosis. In line with this, we also found a positive correlation between mRNA expression of *IL17A* and *COL1A1* in a publicly available human bulk RNA-seq data set (32). Interestingly, cells producing IFN-γ, IL-10, and TNF-α were not enriched in patients with fibrosis, suggesting that these cytokines might be relevant during acute inflammation, but not for the progression to fibrosis. In mice, the inhibition of IL-17A showed reduced mRNA levels of profibrogenic genes, such as *COL1A1*, and a reduced degree of liver fibrosis (29). In different types of chronic liver diseases, such as autoimmune hepatitis (AIH) or alcoholic liver disease, the frequency of IL-17A–producing cells and the expression of Th17-related cytokines were also significantly elevated (44). Furthermore, the duration and severity of hepatitis were suggested to be dependent on Th17 cells in AIH (45). Therefore, all these findings suggest that IL-17A can play a role in the development of chronic liver diseases.

Nevertheless, the overall descriptive nature of our study does not allow us to firmly conclude on a causal link between IL-17A and liver fibrosis in patients. Indeed, one of the main limitations of our study is the lack of functional experiments using, for example, NASH mouse models. These have been developed over recent decades thanks to different types of diets. One of the most commonly used is the methionine/choline-deficient (MCD) diet, which is known to induce NASH-typical histological features in the liver; nevertheless, it has the great limitation of causing weight loss in mice and does not result in the development of insulin resistance and obesity-related comorbidities (46). An alternative to the MCD diet is the Western diet (WD), which mimics the fast-food diet, characterized by high fat, high cholesterol, and high fructose content with and without *trans* fat (47, 48). Unlike the MCD diet, the WD induces the progression to NASH through obesity, but requires a longer feeding time (49). A choline-deficient L-amino acid–defined, high-fat diet (CD-HFD) is a combination of the 2 diets and allows for faster development of NASH yet maintains the obesity-related phenotype (47, 48, 50). These WD-mediated models are most likely the best models at the moment and using these to further test the role of multicytokine-producing CD4^+^ T cells could be considered for future studies.

Another limitation of our study is that, since we decided to focus on memory CD4^+^ T cells, we excluded other cell populations from the scRNA-seq analysis, such as Temra cells, and could not explore their role in the pathogenesis of NASH. Future studies may further investigate the role of these cell subsets in this context.

In conclusion, this descriptive study provides the first single-cell-based atlas to our knowledge of CD4^+^ T cells in NASH and NALFD patients. This resource can be further explored for future studies, but already suggests that targeting multicytokine-producing CD4^+^ T cells can represent a new therapeutic target for NASH patients using, for example, depleting monoclonal antibodies, antibiotics, or probiotic treatments.

## Methods

### Patient samples.

Fresh samples used in the study were obtained from patients undergoing bariatric surgery and simultaneous liver biopsy at the University Medical Center Hamburg-Eppendorf. Corresponding small intestine and liver biopsy samples were obtained during gastric bypass surgery. None of the patients suffered of any diseases of the digestive tract (Supplemental Table 3). Liver samples were 1 × 1 cm and small intestine samples were 3 × 3 cm in size. Histopathological analysis to determine the NAFLD Activity Sore (NAS) and the fibrosis score was performed by a senior specialist in gastrointestinal pathology at the University Medical Center Hamburg-Eppendorf. The NAS score was based on ballooning, inflammation, and steatosis level. Liver and small intestine samples were processed for FACS analysis and scRNA-seq, as described below.

### T cell isolation.

Intraepithelial lymphocytes from the small intestine were incubated in HBSS containing 1 mM dithioerythritol, followed by a dissociation step using 1.3 mM EDTA at 37°C for 30 minutes. Intraepithelial and lamina propria lymphocytes were isolated by digesting the tissue in HBSS (with Ca^2+^ and Mg^2+^) with collagenase (1 mg/mL) and DNase I (10 U/mL) at 37°C for 45 minutes. The cells were further separated using a Percoll gradient (GE Healthcare).

### Flow cytometry.

Briefly, after immune cell isolation using a Percoll gradient, cells were restimulated with PMA/ionomycin and monensin for 3 hours and stained (for antibody panel and clones, see Supplemental Table 4). Samples were acquired on an LSR II flow cytometer (BD Bioscience) and analyzed using Flowjo. Additionally, data were also analyzed using the Cytobank platform (viSNE analysis).

### Real-time PCR.

Total RNA was extracted from liver tissue using TRIzol reagent (Invitrogen). The high-capacity cDNA synthesis Kit (Applied Biosystems) was used for cDNA synthesis. Real-time PCR was performed using the Kapa Probe Fast qPCR Master Mix (Kapa Biosystems) on the StepOnePlus system (Applied Biosystems). Probes used were *HPRT1* (Hs02800695_m1), *CD74* (HS00269961_m1), *COL1A1* (Hs00164004_m1), and *ACTA2* (Hs00909449_m1) (all from Thermo Fisher Scientific). Relative RNA expression was normalized to HPRT and calculated using the 2^−ΔΔCt^ method.

### Immunofluorescence.

Immunofluorescence was performed using standard protocols for human specimens (51, 52); we used a combination of anti-CCR6 (R&D Systems, MAB195-SP) and anti-αSMA (Merk, F3777-.2ML). After immunolabeling, images were obtained using a laser confocal microscope (LSM800, Zeiss).

### In vitro experiment with fibroblasts.

Primary fibroblasts were isolated from the preputium, obtained in a circumcision, from an anonymous donor after confirmed consent of the patient and/or his parents at the dermatology department of the University Medical Center Hamburg-Eppendorf. The tissue was minced and pieces were incubated untouched with RPMI (containing 10% FBS, 1% L-glutamine, and 1% penicillin-streptomycin, all from Gibco) in a 25 cm^2^ cell culture flask (Sarstedt) for 6 days at 37°C and 5% CO_2_. After that, media were changed and cells were trypsinized with Trypsin/EDTA (0.05%/0.02%, Gibco) and passaged at a density of 60%–80% at a rate of 1:3. Cryopreservation was done with cell culture media and 10% DMSO. Cells were thawed, cultured, and passaged via trypsinization upon reaching 80% confluence, according to standard protocols. For the in vitro experiment, 2 mL of a 2.5 × 10^4^/mL HFF (Human Foreskin Fibroblasts) cell suspension in complete RPMI medium were seeded in each well of two 6-well plates and cultured under 5% CO_2_ atmosphere in a standard 37°C humidified CO_2_ incubator. The cells were monitored daily via light microscopy, and upon reaching 80% confluence their media were replaced with 2 mL fresh complete RPMI medium containing 10 ng/mL human TGF-β1 and 150 ng/mL human IL-17A in duplicate. Two wells containing 80% confluent HuFu cells were also left untreated. The cells were incubated with the cytokines for an additional 24 hours under 5% CO_2_. Next, the cells were washed twice with PBS, scraped from the plate using a cell scraper, and centrifuged at 500*g* for 5 minutes at 4°C using a table-top microcentrifuge. The supernatant was discarded and the cell pellets were lysed with TRIzol and passed through a Qiagen RNeasy mini column followed by on-column DNase digestion (to remove genomic DNA) for total RNA purification. RNA samples (0.5 μg each) were subjected to first cDNA synthesis with oligoDT primer using the RevertAid H minus First-Strand cDNA Synthesis Kit (Thermo Fisher Scientific) according to the manufacturer’s instructions in a volume of 20 μL. Next, the first-strand synthesis reaction was diluted with sterile H_2_O to 100 μL total volume and 2 μL of each reaction was used as a template for qPCR using Taqman Gene Expression Master Mix (Applied Biosystems) in a total reaction volume of 10 μL, using the following Taqman probes: *ACTA2* (Hs00426835_g1), *COL1A1* (Hs00164004_m1), *HPRT1* (Hs03929098_m1), *TGFB1* (Hs0098133_m1), and *TBP* (Hs00427620_m1) (all from Thermo Fischer Scientific), in a 384-well Image QuantStudio qPCR controller (Applied Biosystems) in standard Taqman reactions. The results were exported from the controller as.xls files and the raw data were analyzed in Microsoft Excel spreadsheets using the 2^–ΔΔCt^ method. The whole experimental procedure was performed in triplicate. The analyzed data are presented herein as *z* scores.

### scRNA-seq: cDNA library preparation.

The sorted cellular suspension was loaded on a 10× Genomics Chromium instrument to generate single-cell gel beads in emulsion (GEMs). scRNA-seq libraries were prepared as described by the 10× Genomics Single Cell 3′ v2 Reagent Kit user guide and using the following reagent kits: Chromium Single Cell 3′ Library & Gel Bead Kit v2 (PN-120237), Chromium Single Cell A Chip Kit (PN-120236), and Chromium i7 Multiplex Kit (PN-120262). Data were uploaded to the NCBI Gene Expression Omnibus database (GEO GSE217235).

### scRNA-seq: preprocessing and quality control.

10× Genomics raw sequencing data were processed using CellRanger software (v2.1.0, 10× Genomics) and the 10× human genome GRCh38 1.2.0 release as the reference (function cellranger count). The matrices of cells and the unique molecular identifier (UMI) count were obtained and further processed by the R package Seurat (v3.0.2) (53). In order to avoid potential contamination of non–T cells during sorting, only cells with an average count of CD3D, CD3E, and CD3G higher than 0 were kept for further analysis.

### scRNA-seq: dimensionality reduction and clustering.

To reduce sample batch effects, we applied the integration method implemented in Seurat v3 (functions FindIntegrationAnchors and IntegrateData, dims = 1:30). The integrated matrix was then scaled with the ScaleData function (default parameters). Principal component analysis (PCA) was performed on the scaled data (function RunPCA, npcs = 30) to reduce dimensionality. The selected principal components were then used to compute the KNN graph based on the Euclidean distance (function FindNeighbors). Cell clusters were subsequently generated using the function FindClusters. The resolution of the FindClusters function was determined by exploration of the top marker genes of each cluster.

The Seurat R package (v3.0.2) was used to perform unsupervised clustering analysis on scRNA-seq data. t-SNE was used to visualize clustering results. The top differentially expressed genes in each cluster were found using the FindAllMarkers function (min.pct = 0.25, logfc.threshold = 0.25) that ran Wilcoxon’s rank-sum tests. The differential expression between conditions (NASH vs. NAFLD) were calculated by the FindMarkers function (min.pct = 0.1), which also ran Wilcoxon’s rank-sum tests.

### scRNA-seq: cell type annotation.

We determined the cell populations by examining the most differentially expressed genes of each population and comparing our data set with standard markers used in flow cytometry and already existing sequencing data of cells in the liver (15–18). For Trm and Tr1 cells, which have less-well-defined markers, we generated scores based on different gene sets. Average expression levels of the Trm gene set (Trm score) and Tr1 gene set (Tr1 score) (Supplemental Table 5) were calculated using Seurat function (24) AddModuleScore (nbin = 24, ctrl = 100). Trm signature genes were obtained from the study of sorted CD69^+^CD4^+^ and CD69^–^CD4^+^ T cells of the lung, spleen, and blood (26). Tr1 signature genes were first selected from the study of mouse Tr1 cells (16) and then converted to corresponding human genes using the getLDS function from the biomaRt package (v2.40.3; https://bioconductor.org/packages/release/bioc/html/biomaRt.html).

### scRNA-seq: trajectory analysis.

The slingshot R package (v1.3.2) (54) and the Monocle 3 R package (v1.2.9) (55) were used for trajectory analysis. For the slingshot analysis, the Seurat objects of the selected clusters were first converted to SingleCellExperiment object (function as.SingleCellExperiment). Then, function slingshot (reducedDim = “PCA”) was used to identify the global lineage structure with a cluster-based minimum spanning tree (MST) and fit simultaneous principal curves to describe each lineage. The pseudotime of each cell was also obtained in this step and then visualized by ggplot R package (v3.2.1). For the Monocle analysis, the function learn_graph (default parameters) was used to fit a principal trajectory within the t-SNE plot of the cells.

### Integrated analysis with external scRNA-seq data.

The scRNA-seq data set (19) was downloaded from NCBI GEO via accession number GSE136103. The sample integration, dimensionality reduction, clustering, and cell type annotation methods were the same as described above. The CD4^+^ T cell count matrices were extracted using the subset function after integration and clustering with Seurat R package (v3.0.2).

### Correlation analysis of external bulk RNA-seq data.

The human liver bulk RNA-seq data set (31) was downloaded from the NCBI BioProject via accession number PRJNA512027. The raw reads were aligned to the human reference genome (GRCh38) using the RSEM (v1.3.0) pipeline with default parameters. The function rsem-calculate-expression was used to align the reads and quantify the gene and isoform abundance. The output of rsem-calculate-expression separately gives the read count and transcripts per million (TPM) value for each gene and isoform. The correlation of TRM counts from samples were calculated in R (v4.1.1) with cor.test function.

### Random forest.

To determine the set of most discriminative features, we used a random forest binary classification algorithm, implemented in the randomForest R package (v4.6-14). Performance measures like AUC, sensitivity, specificity, recall, and precision were calculated using caret (v6.0-79) and ROCR (v1.0-7) R packages. The analysis was performed using R v3.4.1.

### 16s rRNA gene metagenomic sequencing of liver and small intestine tissues.

First, a host DNA depletion by osmotic lysis in distilled water, followed by PMA treatment was conducted according to Marotz et al. (56). Next, bacterial DNA was extracted using the PowerMag Microbiome DNA isolation kit (Qiagen) following the manufacturer’s instructions. DNA libraries were constructed, and high-throughput sequencing was performed on a MiSeq platform (Illumina). A negative control was used to test the potential presence of contaminant DNA; the sample showed no product.

### 16S rRNA sequence data analysis.

Raw fastq files were checked for overall data quality using FastQC (v0.11.5; https://www.bioinformatics.babraham.ac.uk/projects/fastqc/). All further preprocessing and analysis steps were performed in R (v3.6.0).

We used the dada2 (57) (v1.12.1) package to generate amplicon sequence variants (ASVs) by following the authors recommended processing steps for paired sequencing data. In brief, all reads were first run through quality filtering using the fastqPairedFilter function with default options, except for the number of truncated base pairs per read where we used 180 bp and 150 bp, respectively. Additionally, reads with more than 2 expected errors were removed. On average, 67.8% of input reads were kept after all filtering steps. Lastly, we removed any ASVs that were observed in fewer than 1% of the samples.

Taxonomic assignment was performed using the DECIPHER (58) (v2.12.0) package against the SILVA SSU database (release 132; https://www.arb-silva.de/documentation/release-132/; accessed March 2018). All further analysis was performed within the phyloseq framework (v1.32.0). Due to widely differing sequencing depth and subsequently differing number of ASV counts (4–15.757; median, 557) we decided to rarefy our data. After inspecting rarefaction curves we filtered out all samples with fewer than 500 counts and rarefied all remaining samples to an ASV count of 500.

To perform the linear discriminant analysis effect size (LEfSe) we used proportional abundances at the genus level and tried normalization factors of 10,000 and 1,000,000 in the LEfSe format_input step.

Alpha diversities were calculated using the estimate_richness function. Bray-Curtis distances were calculated using the distance function and differences between the resulting distance matrices were analyzed using PERMANOVA with the adonis function from the vegan package (v2.5.6; https://cran.r-project.org/src/contrib/Archive/vegan/vegan_2.5-6.tar.gz), again using categorized age and BMI covariates. Differential abundance in ASV counts was assessed using the metagenomeSeq package (59) (v1.30.0). The differential abundance threshold was set to FDR less than 0.05 and an absolute log(fold change) (logFC) greater than 0.5.

### Statistics.

For nontranscriptomic data, 2-tailed Mann-Whitney *U* test, Wilcoxon’s test, unpaired *t* test with Welch’s correction, or 1-way ANOVA with Tukey post hoc test was used to calculate significance, where applicable. Statistical calculations were performed using Prism 5.0 (GraphPad Software). Statistical significance was set to *P* less than 0.05.

### Study approval and ethics statements.

The study was approved by the Ethics Committee of Hamburg and the study protocol was conformed to the ethical guidelines of the 1975 Declaration of Helsinki. Written informed consent was obtained from all patients and experiments were carried out in accordance with all relevant ethical regulations.

## Author contributions

AW designed the research studies, conducted experiments, acquired data, and wrote the manuscript. PS designed the research studies, conducted experiments, acquired data, and wrote and revised the manuscript. YZ designed the research studies, analyzed data, and wrote and revised the manuscript. JW, FM, ADG, MNW, AKM, AAB, FS, FC, SM, AS, TA, LK, and RW conducted experiments and acquired data. CC, VB, and TZ analyzed data. KFK acquired data. AD, SW, OM, and JRI provided samples. VGP, SB, SH, and AWL designed the research studies. TBH provided cell lines. NWP provided access to the method to analyze the microbiota. NG designed the research studies, wrote the manuscript, and supervised the project. AW, PS, and YZ designed the study together and cooperated to analyze the data, contributing equally to the development of the project.

## Supplementary Material

Supplemental data

Supplemental table 1

Supplemental table 2

Supplemental table 3

Supplemental table 4

Supplemental table 5

## Version 1 01/10/2023

Electronic publication
