# Supplementary material for: Multicytokine-producing CD4+ T cells characterize the livers of patients with NASH
Source: JCI Insight. 2023 Jan 10;8(1):e153831. doi: 10.1172/jci.insight.153831 (PMC9870087; doi:10.1172/jci.insight.153831)
Supplement: Supplemental data [file jciinsight-8-153831-s110.pdf]

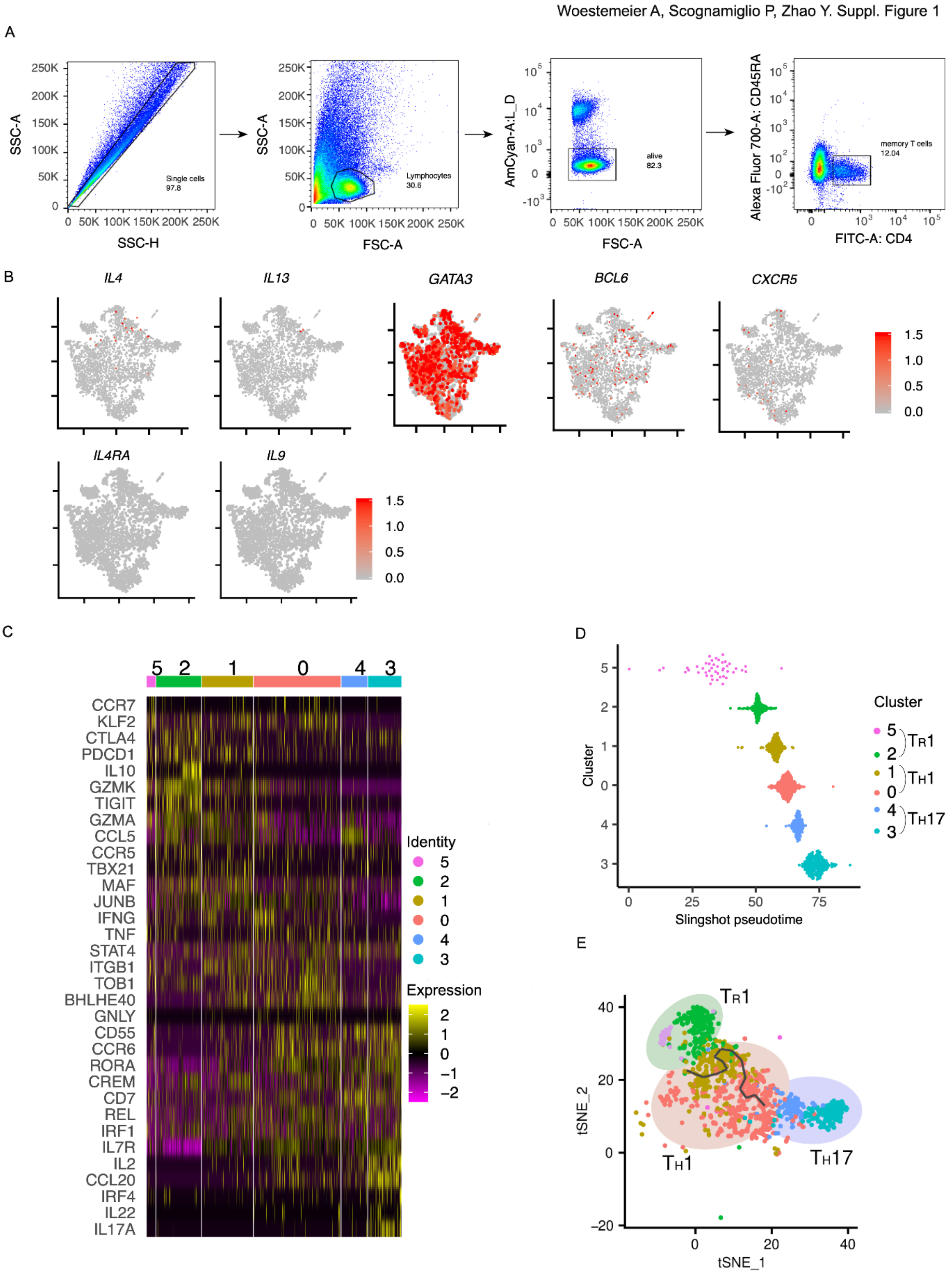

Suppl. Figure 1 ScRNA-seq of CD4⁺ T cells found in the liver of NAFLD patients | (A) Sorting strategy of CD4⁺ CD45RA⁻ cells scRNA-seq from NAFLD patients. **(B)** tSNE maps reporting the cells expressing the indicated signatures genes of T_H2, T_H9 and T_{FH} cells. **(C)** 1312 cells were subclustered. Heat map depicted the average expression levels per subcluster of the most differentially expressed genes. (**D)** Slingshot pseudotime analysis of the reported CD4⁺ T cell subclusters **(E)** Monocle pseudotime analysis of the reported CD4⁺ T cell subclusters.

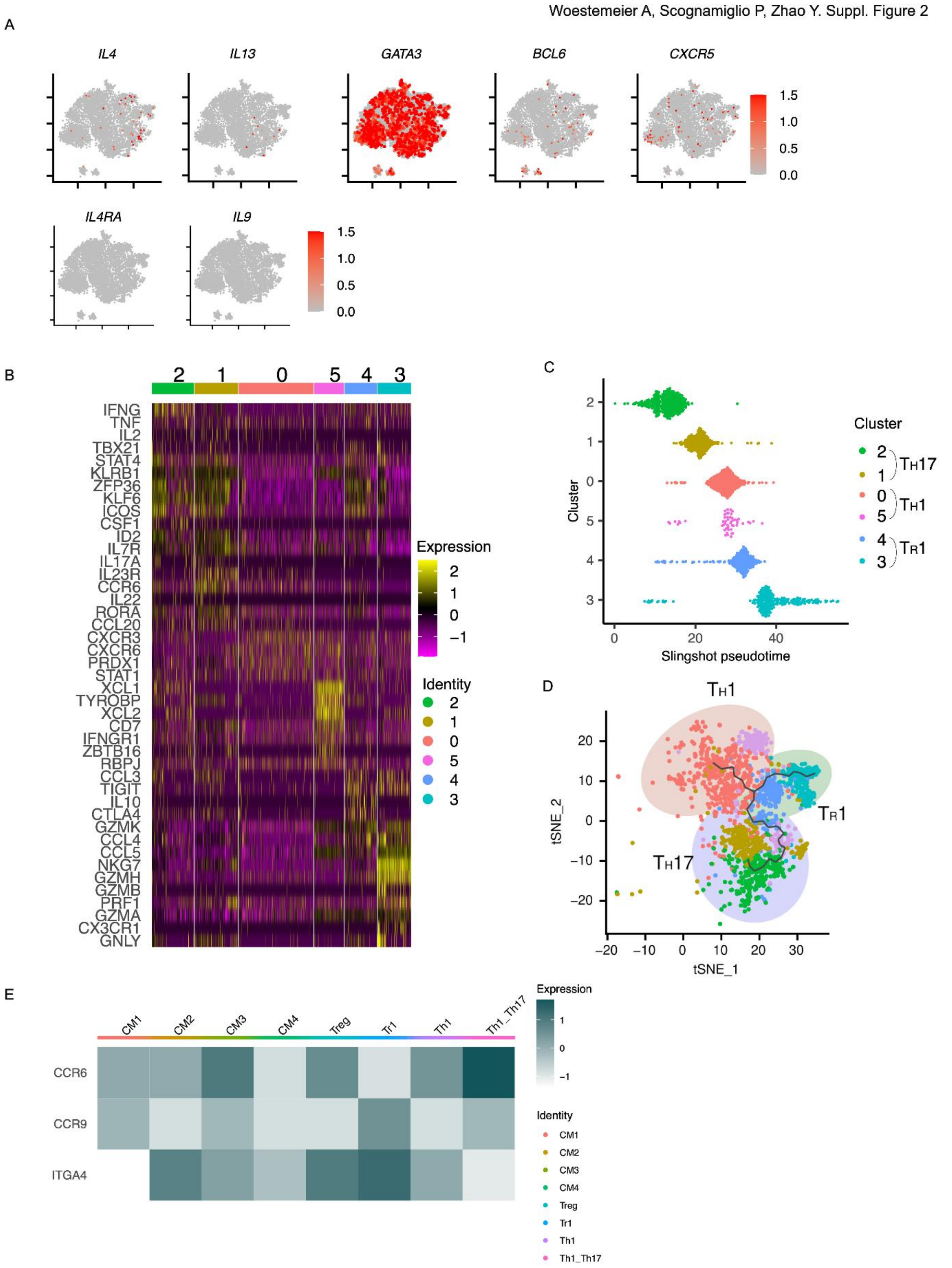

Suppl. Figure 2 | ScRNA-seq of CD4+ T cells found in the liver of NASH patients (A) tSNE maps reporting the cells expressing the indicated signatures genes of T$_{H}$2, T$_{H}$9 and T$_{FH}$ cells form NASH patients. **(B)** 1898 cells were subclustered. Heat map depicted the average expression levels per subcluster of the most differentially expressed genes. **(C)** Slingshot pseudotime analysis of the reported CD4^{+} T cell subclusters. **(D)** Monocle pseudotime analysis of the reported CD4^{+} T cell subclusters. **(E)**

Heatmap of the expression of the indicated chemokine and integrin genes of the reported $CD4^{+}$ T cell subclusters in the liver of NASH patients.

Woestemeier A, Scognamiglio P, Zhao Y. Suppl. Figure 3

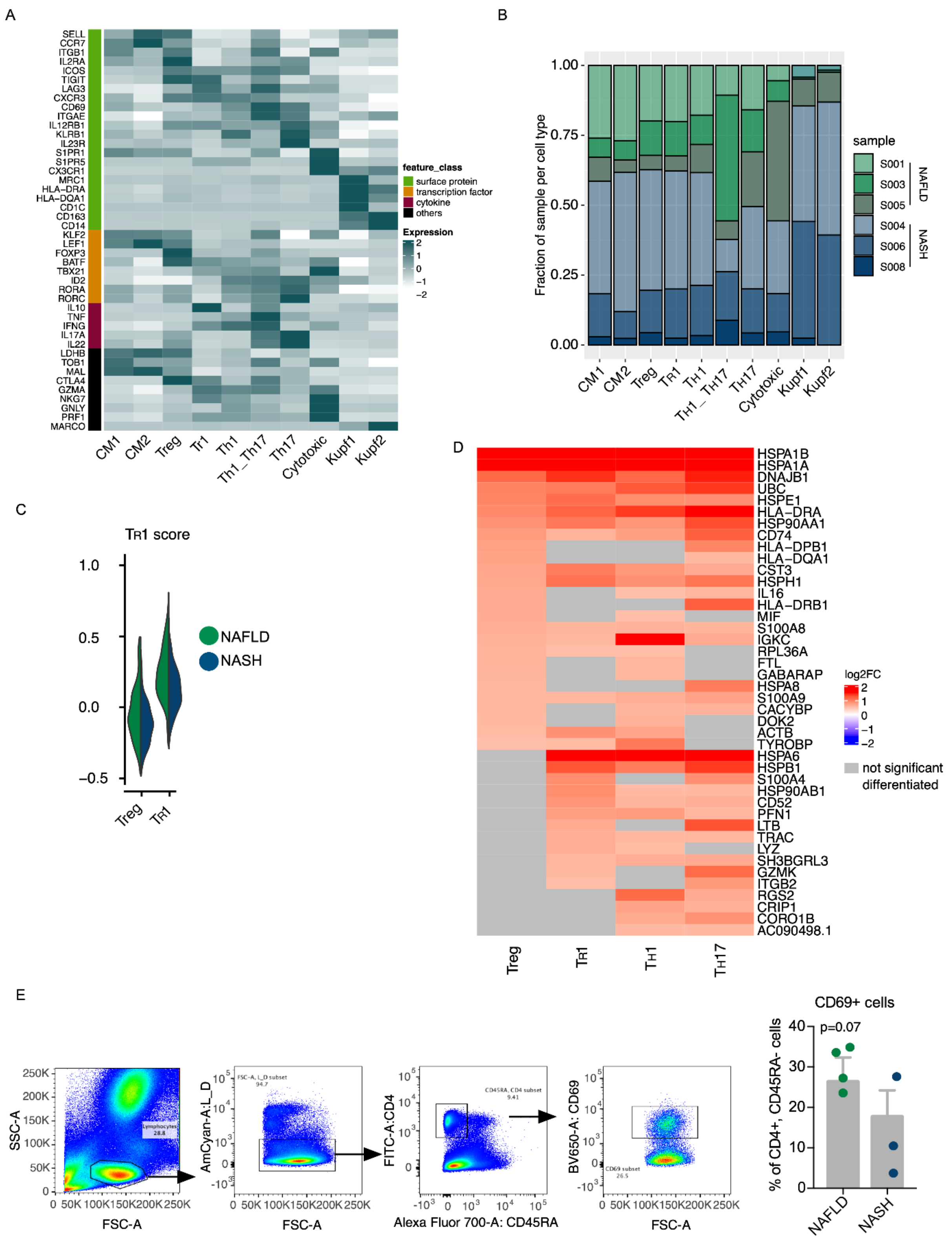

Suppl. Figure 3 | Differences between CD4+ T cells in the livers of NAFLD and NASH patients (A) Heatmap of CD4+ T cell clusters displaying key signature genes to annotate the different cluster **(B)** Distribution of each cell clusters for each NAFLD and NASH patients. **(C)** Expression of the TR1 gene signature score of the indicated clusters of human CD4+ T cells in NAFLD and NASH. **(D)** Heatmap showing the differential RNA expression analysis across T cell clusters. **(E)** Frequencies of CD69+ cells within CD4+ CD45RA- T cells (right). Each dot represents a patient. Data are presented as mean ± SEM.

P.value was determined by Mann–Whitney U test. **(F)** Heatmap of the expression of the indicated chemokine and integrin genes of the reported $CD4^+$ T cell subclusters in the liver of NASH patients.

Woestemeier A, Scognamiglio P, Zhao Y. Suppl. Figure 4

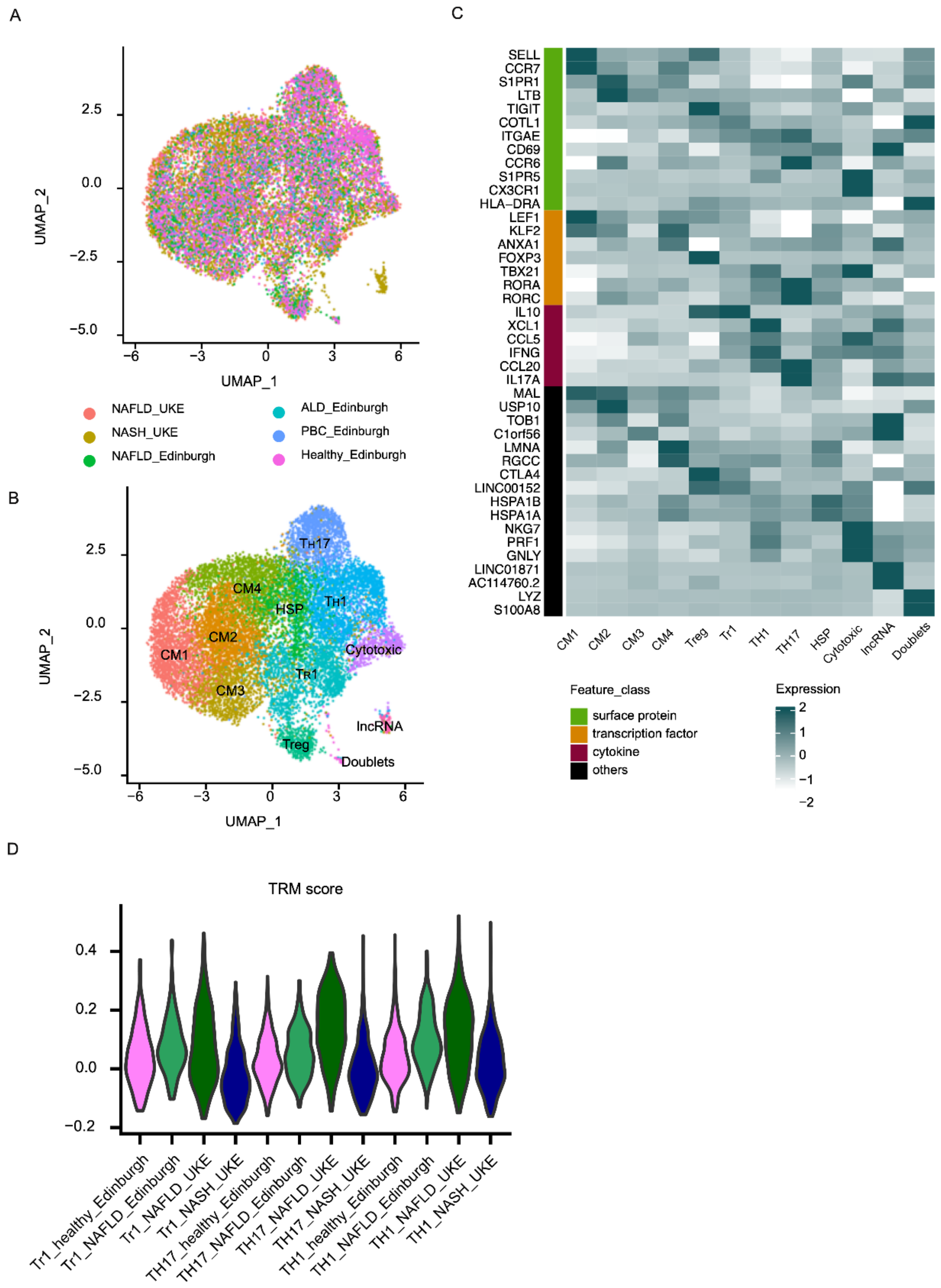

Suppl. Figure 4 | Integrated analysis of our data with the data from the study from Ramachandran P et al (19), comparing NALFD and NASH patients with healthy patients. (A) t-SNE plot of CD4⁺ T cells from our (UKE) scRNA-seq data set merged with a published date set (Edinburgh). **(B)** Clustering and cell type annotation of integrated CD4⁺ T cells from UKE and Edinburgh datasets. **(C)** Heatmap of CD4⁺ T cell clusters showing the most differentially expressed genes and other key signature genes used to annotate the cells. **(D)** Expression of the T_{RM} gene signature score of the indicated clusters of

human $CD4^+$ T cells in the livers of healthy controls (from Edinburgh) and NAFLD (from Edinburgh and UKE) and NASH (from UKE) patients.

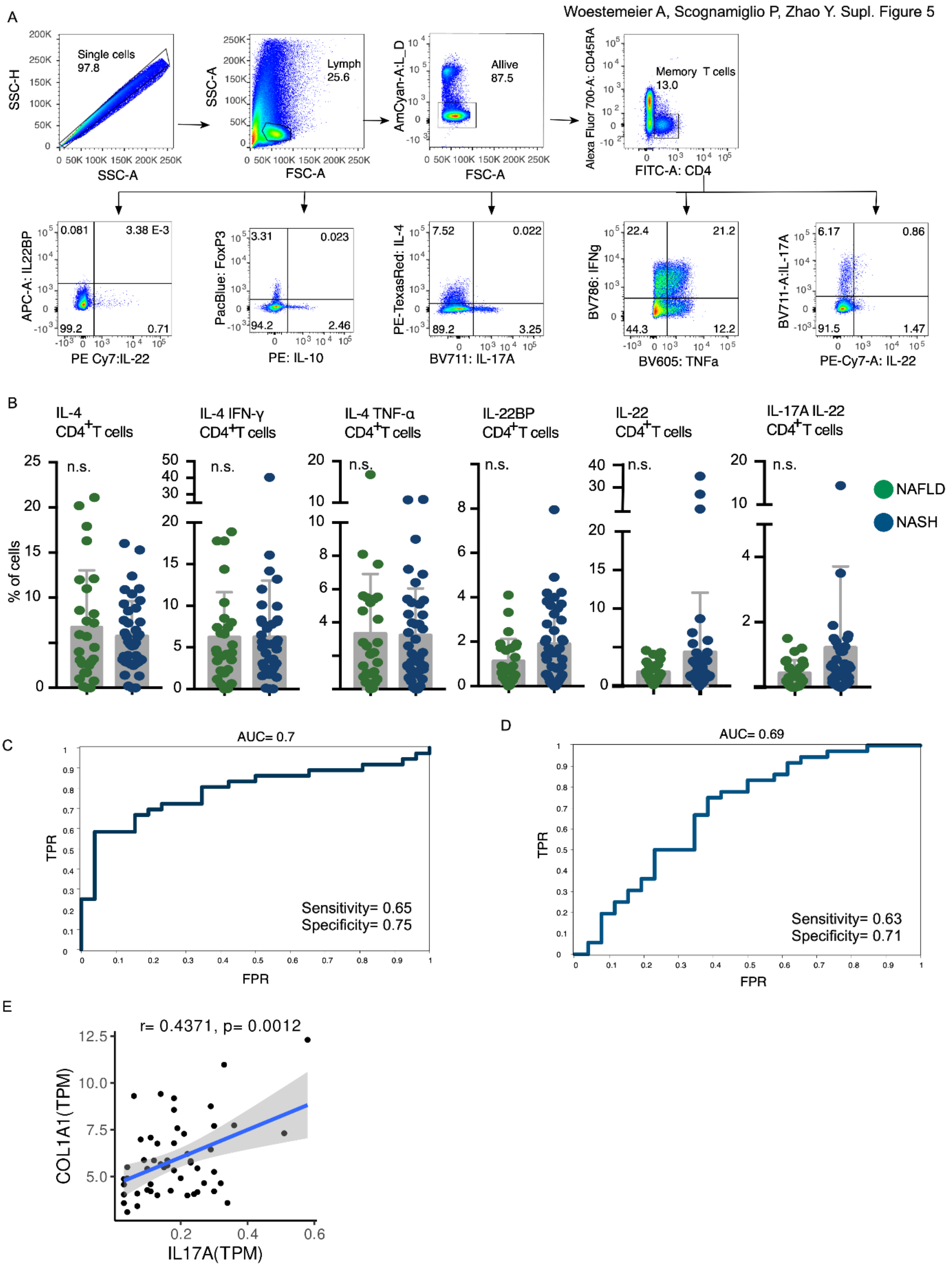

Suppl. Figure 5. | Flow cytometry analysis of liver CD4⁺ T cells, ROC curves and correlation between *IL17A* and *COL1A1* analyzed using a publicly available human NASH bulk RNA-seq data set(32) (A) Representative gating strategy of the flow cytometry analysis of stimulated liver samples used for the quantification of cytokine production. **(B)** Frequencies of the indicated liver multi-cytokine-producing CD4⁺ T cells. Each dot represents a patient. Data are presented as mean ± SEM. P.values were determined Mann–Whitney U test. N.s.: non significant (p>0.05). **(C)** ROC curve showing true- and false-positive rates for the discrimination between NAFLD and NASH prediction based on sex, age, BMI,

transaminase levels. (**D)** ROC curve showing true- and false-positive rates for the discrimination between NAFLD and NASH prediction based only on cytokine levels. **(E)** Pearson correlation of *IL17A* and *COL1A1* (32). The linear regression is depicted by the blue lines, the grey shades show confidence intervals. Each dot represents a patient. Pearson Correlation Coefficient r and p value are reported. Gene expression level is calculated based on average TPM (transcript per million) of each sample.

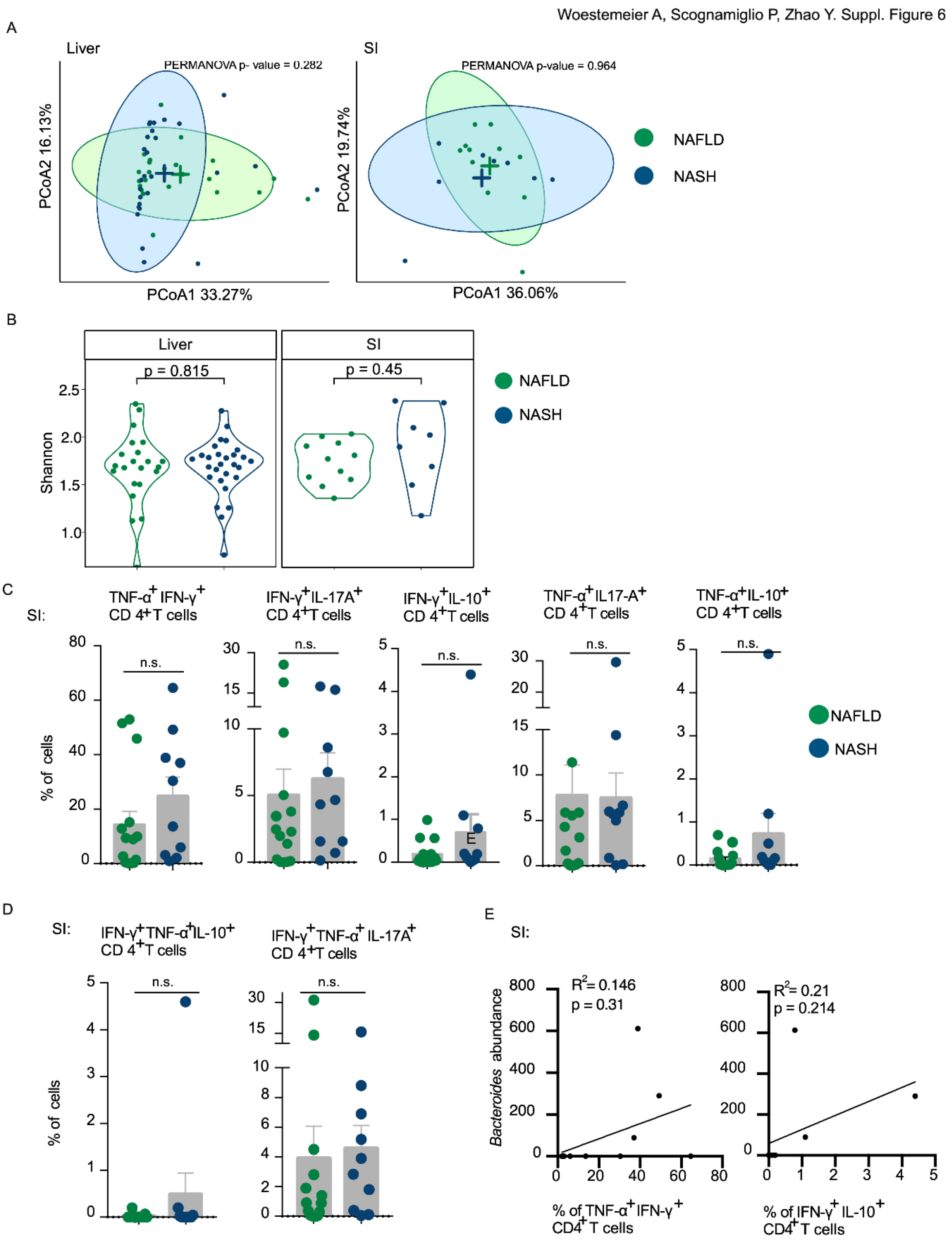

Suppl. Figure 6 | Characterization of the liver and intestinal microbiota and cellular characterization of the small intestine. (A) Differences of microbial β-diversity between NAFLD and NASH patients within liver and small intestine (SI) samples. Β-diversity was visualized by constrained analysis of principal coordinates using Bray-Curtis distance. Each dot represents a patient. P Value was calculated with the PERMANOVA test. **(B)** Violin plots for Shannon α-diversity within liver and SI tissues. Each dot represents a patient. P. value were determined by fitFeatureModel from the R package metagenomeSeq. **(C)** Frequencies of the indicated multi-cytokine-producing CD4+ T cells isolated from

the small intestine of NAFLD and NASH patients undergoing bariatric surgery. Each dot represents a patient. Data are presented as mean ± SEM. P.values were determined by Mann–Whitney U test. N.s.: non-significant (p>0.05). **(D)** Frequencies of the indicated multi-cytokine-producing CD4+ T cells isolated from the small intestine of NAFLD and NASH patients undergoing bariatric surgery. Each dot represents a patient. Data are presented as mean ± SEM. P.values were determined by Mann–Whitney U test. N.s.: non-significant. (p>0.05). **(E)** Correlation between Bacteroides abundance in the small intestine and frequency of the indicated cytokine-producing $CD4^+$ T cell subsets in the small intestine of NAFLD and NASH patients. Each dot represents a patient. P values were calculated with the Pearson's correlation coefficient.

Table legend

Supplementary Table 1: clinical and demographic data of patients included in the SCS analysis

Supplementary Table 2: Integration Anchors, raw data

Supplementary Table 3: Clinical and demographic characteristics and cytokine production of patients included in the FACS analysis

Supplementary Table 4: Antibody panel and clones for the FACS Analysis

Supplementary Table 5: Gene set for TRM and Tr1
